# A rare case of multiple supratentorial brain lesions due to meningiomatosis

**DOI:** 10.1016/j.radcr.2023.08.037

**Published:** 2023-09-04

**Authors:** R. Vera Indriani, Gustiara Munir, Birgitta M. Dewayani

**Affiliations:** a Department of Radiology, Faculty of Medicine, Hasan Sadikin General Hospital, Padjadjaran University, Jl. Pasteur No.38, Pasteur, Bandung, West Java, 40161 Indonesia; b Department of Pathology Anatomy, Faculty of Medicine, Hasan Sadikin General Hospital, Padjadjaran University, Jl. Pasteur No.38, Pasteur, Bandung, West Java, 40161 Indonesia

**Keywords:** CT scan, Meningiomas, Meningiomatosis, MRI

## Abstract

Meningeal tumors represent the most common primary central nervous system tumors. The term “multiple meningiomas” or “meningiomatosis” refers to the occurrence of 2 or more spatially separated meningiomas without the features of neurofibromatosis. Meningiomatosis accounts for only less than 10% of all cases and is more prevalent in women. We report a rare case of a 53-year-old female patient complaining of a headache characterized by a throbbing pain in the right side of the head. Neurological examination was largely normal, with the exception of a slight weakening of the right extremity. Multiple brain masses, due to meningiomatosis, were revealed upon CT scan and MRI. Subsequent tissue biopsy confirmed the diagnosis of meningothelial meningiomas.

## Introduction

Meningeal tumors are the most common primary central nervous system tumors. They consist of both meningiomas and nonmeningeal mesenchymal tumors. Although most are attached to the dura mater, they can occur in other locations, such as the choroid plexus [1]. The term “multiple meningiomas” or “meningiomatosis”—coined by Cushing and Eisenhardt—refers to the occurrence of 2 or more spatially separated meningiomas without fibromatosis [2]. Among all primary intracranial and central nervous system tumors, meningiomas have the highest incidence rate at 37.6%. The incidence rises with age, especially after 65 years [3]. Meningiomatosis is found in less than 10% of patients with meningiomas, and it is more prevalent in women, with a female-to-male ratio of 3.5:1 [4]. In this case report, we present a rare case of multiple supratentorial brain lesions attributable to meningiomatosis.

## Case report

A 53-year-old female presented with a history of progressively worsening headaches for approximately 3 months, which had intensified markedly 2 days before her admission to the hospital. The headaches were characterized by a throbbing sensation on the right side of her head. Patient had neither experienced seizures nor reported any weakness in her limbs. Throughout this period, the patient remained conscious, could understand speech, and could follow commands. The patient had a 2-year history of hypertension, which had been well-managed. Physical examination, including assessment of vital signs, presented normal results.

**Fig. 1:**
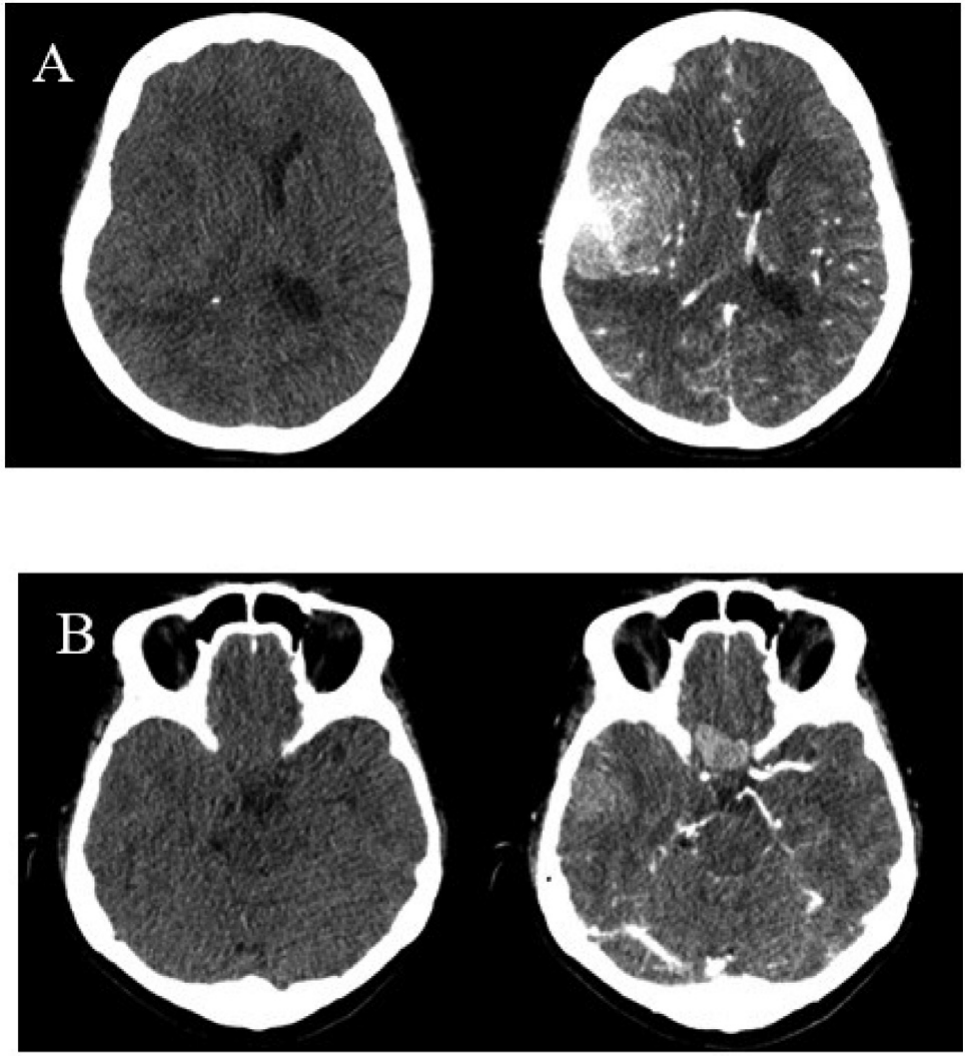
Head CT scan axial section showed inhomogeneous isodense mass in the frontotemporoparietal region dextra and extending into the surrounding (A), an inhomogeneous isodense mass in the right temporalis and suprasellar region (B), enhanced after contrast administration (A, B).

The neurological examination was largely unremarkable, except for a slight weakness in the motor function of her right extremity. Her laboratory tests showed an elevated leukocyte count. Computed tomography (CT) scans, performed both with and without contrast, revealed multiple solid, noncalcified masses in the suprasellar region and the right frontotemporal parietal region. These masses had extended to the subcortical cortical part of the right frontotemporal parietal region and were accompanied by edema. Hyperostosis of the calvaria os frontotemporoparietalis sinistra and dextra was also observed. A mass was present in the right frontotemporal parietal region (frontotemporoparietal dextra), applying pressure and causing constriction of the surrounding sulcus gyrus, fissure sylvii dextra, ventricle lateralis dextra and leading to a midline shift towards sinistra (Fig. 1).

**Fig. 2:**
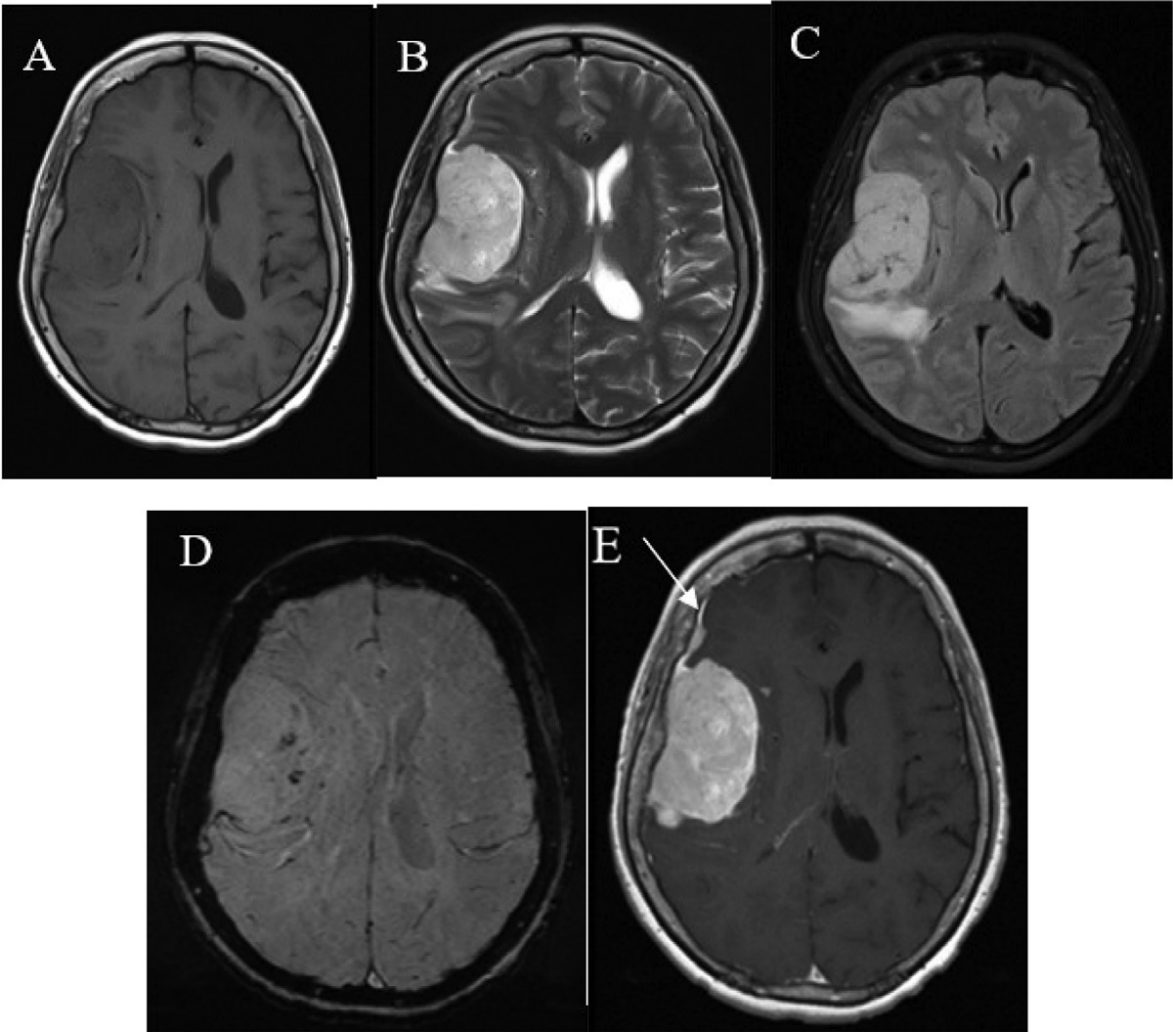
MRI showed hypointense mass in T1W1 (A), hyperintense frontotemporoparietal dextra region mass in T2W1(B), hyperintense mass in T2-FLAIR (C), blooming artifacts in SWI (D), T1+ Contrast showed enhanced mass, *dural tail sign* (white arrow) (E).

**Fig. 3:**
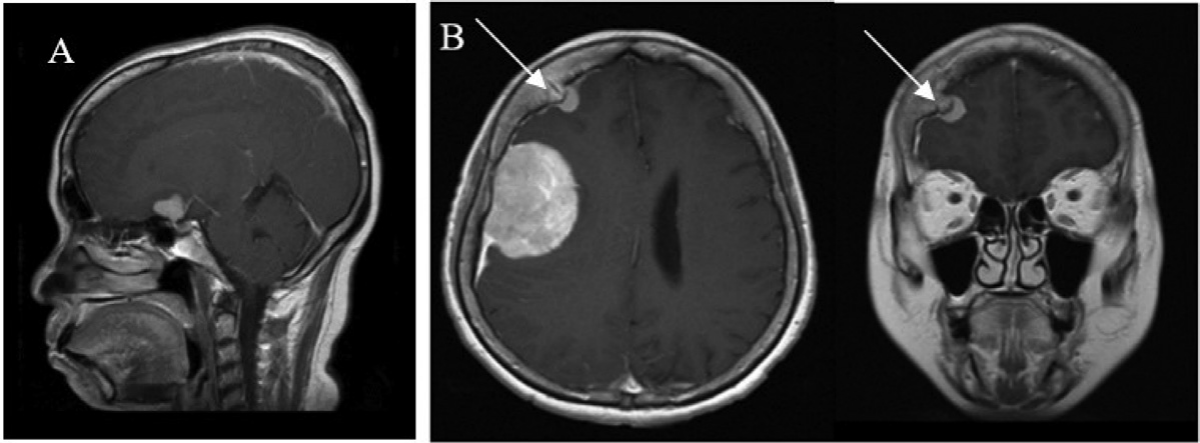
MRI meningiomatosis. (A) T1+ gadolinium contrast sagittal section showing mass in the suprasellar. (B) T1+ gadolinium contrast axial and coronal section showing hyperostosis of os frontalis dextra (white arrow).

Magnetic resonance imaging (MRI) examination of the head revealed multiple masses with a “dural tail” sign (Fig. 2), filling the right frontotemporal parietal concavity (frontotemporoparietal concavity dextra) and the suprasellar region (Fig. 3), accompanied by surrounding perifocal edema. Hyperostosis was observed in the right frontotemporal and left frontal bones (frontotemporoparietal os dextra, os frontalis sinistra). No restricted areas were observed in diffusion weighted imaging with an apparent diffusion coefficient value of 1.2×10^-3^ mm^2^/s and blooming artifacts in susceptibility weighted imaging. Magnetic resonance spectroscopy (MRS) displayed levels of increased glutamine, choline, along with levels of decreased N-acetyl aspartate and creatinine, consistent with the presence of meningiomas.

**Fig. 4:**
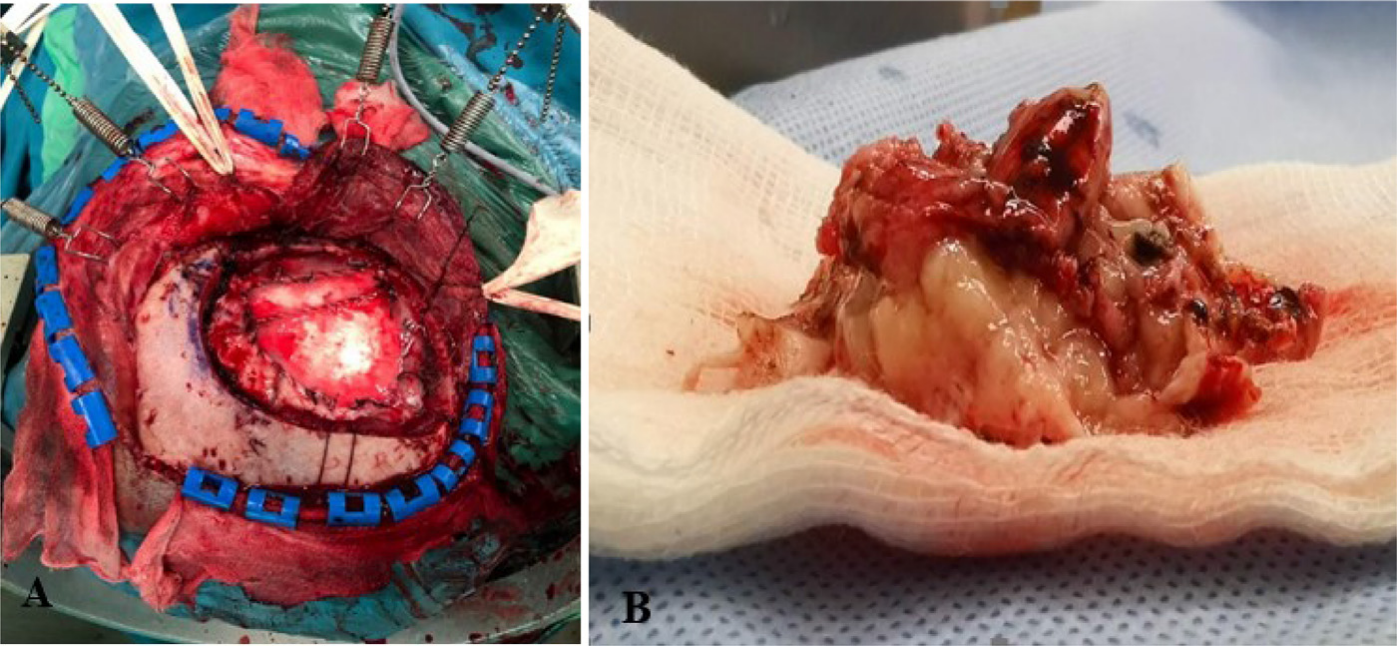
Craniotomy was performed on the patient under general anesthesia (A), a cut section of the tumor (B).

**Fig. 5:**
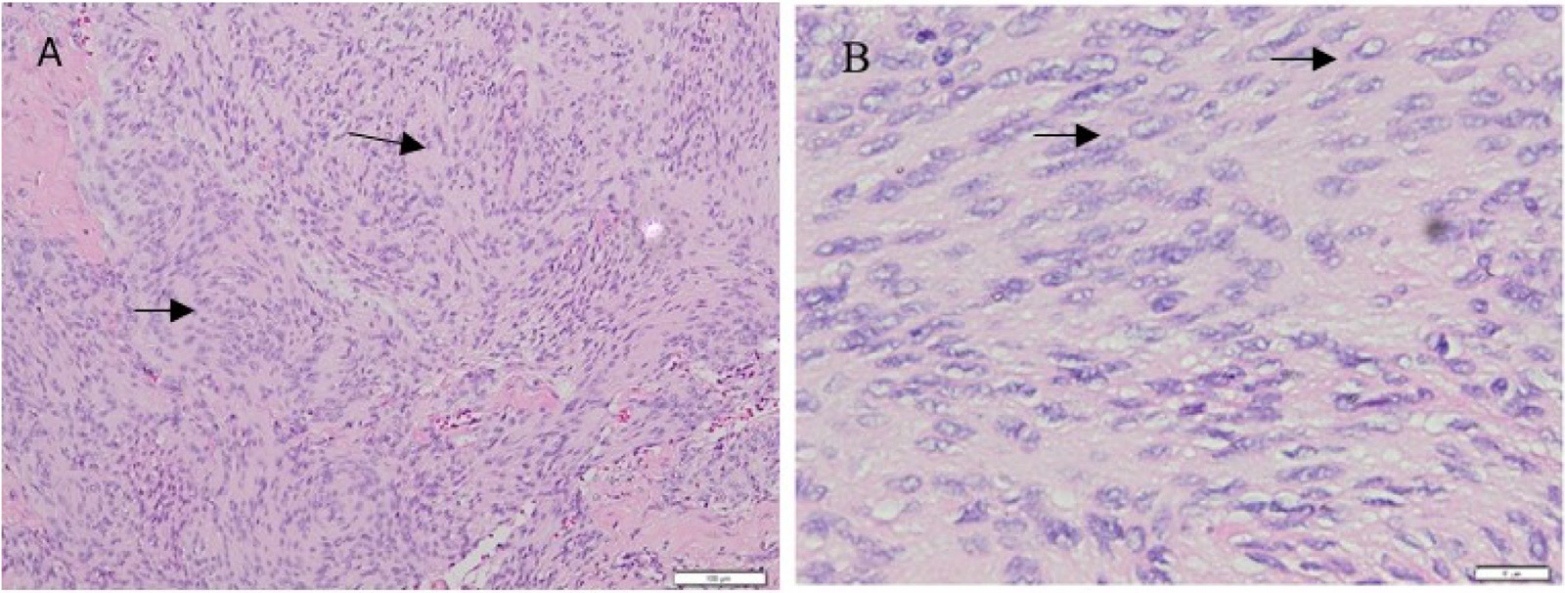
Histological features of meningothelial meningiomas. (A) Largely monomorphic cells with abundant eosinophilic cytoplasm and are rarely arranged in lobules demarcated by fine collagen septa (arrow) (Hematoxylin Eosin, 100×). (B) Borders between the cells are hardly appreciable. The round to oval nuclei has internal empty spaces (nucleus holes) and pseudo-inclusions (arrow) (Hematoxylin Eosin, 400×).

A postsurgical assessment revealed hyperostosis of the bone, tumor-infiltrated duramater, and a greyish white, easily bleeding mass (Fig. 4). Approximately 95% of the tumor was successfully removed. A histopathological examination of the tissue biopsy confirmed the diagnosis of meningothelial meningiomas (Fig. 5).

## Discussion

Meningiomas, originating from arachnoid cap and meningothelial cells, are typically found within the arachnoid layer of the meninges or pacchionian granulations and typically demonstrate dural attachment [4], [5], [6]. The term “multiple meningiomas” or “meningiomatosis” is used when more than 2 meningiomas are present simultaneously at different locations [7]. The average onset age for typical meningiomas is approximately 55 years, with the incidence increasing with advancing age. The morbidity rate of meningiomas in women is higher compared to the incidence in men [8].

According to a study by Cai et al., the main symptom of meningiomas was seizures reported in 65.8% or 25 patients. Other reported symptoms included headache (39.5%, or 15 patients) and hemiparesis (18.4%, or 7 patients). Less frequent early symptoms included visual disturbances (2.6%, or 1 patient), incidental (2.6%, or 1 patient), and dizziness (2.6%, or 1 patient). The incidence of seizures in patients with meningiomas located at the Sylvian fissure (65.8%) was significantly higher than in patients with generalized supratentorial meningiomas (29.2%). Generally, the frequency of seizures appears to be related to the tumor's location, with the temporal and limbic lobes having the lowest threshold for seizure generation. Moreover, hypoxia and metabolic imbalances caused by tumor invasion and extrusion are considered influential etiological mechanisms [5].

As the brain tumor grows, it progressively replaces healthy tissues, leading to vascular abnormalities and blood-brain barrier disruption. This results in substantial blood plasma leakage into the tumor tissue, causing cerebral edema and abnormal swelling of the cerebral parenchyma. Given that the skull is a closed compartment, any growth of the tumor and its associated cerebral edema can induce increased intracranial pressure (ICP) and create a space-occupying lesion. An space-occupying lesion refers to the occupation of intracranial space due to an increase in volume. Elevated ICP is responsible for many adverse symptoms associated with brain cancer, such as headaches, nausea, and seizures [9]. Meningiomas can also cause headaches as an early symptom or during the journey of the disease in most cases. Headache can occur even with tumors of small size, possibly due to dural irritation [10].

Meningiomas are classified by the World Health Organization (WHO) into 3 categories: benign (WHO Grade I), atypical (WHO Grade II), and malignant (WHO Grade III). This classification is based on criteria such as the number of mitoses, the degree of anaplasia, and the presence of necrosis [11]. In terms of frequency, meningotheliomatous meningiomas are the most common subtype, accounting for 70.03% of cases. This is followed by the fibrous variant (7.58%) and the psammomatous type (5.77%). Moreover, 90.22% of all diagnosed cases fall under the classification of WHO Grade I meningiomas [12].

Plain radiographic findings of brain tumors may reveal signs of increased ICP (eg, erosion of the lamina dura of the dorsum sellae or a “J”-shaped sella), tumor calcification, or enlargement of the middle artery indentation in meningiomas [13]. In certain cases, the combined use of CT scans, and MRI can enable a high-probability diagnosis of intracranial meningiomas [14]. On noncontrast CT scans, almost three-quarters of meningiomas appear mild to moderately hyperdense compared to the cortex, while the remains typically display isodensity. Hypodense meningiomas do occur but are relatively rare. Over 90% of meningiomas present as solid, hyperdense masses, with a marked increase in density following intravenous contrast administration. Approximately 25% of these tumors exhibit calcifications, and the underlying skull often displays hyperostotic [1,15]. CT scans with a bone window setting effectively assess hyperostosis in adjacent bone and intraosseous tumor growth [3,16].

MRI is the gold standard for advanced imaging of meningiomas. The size of meningiomas is typically evaluated using a T1 sequence complemented by a gadolinium injection [3,17]. On MRI examination of supratentorial meningiomas, approximately 85%-90% present as a well-circumscribed frontoparietal extra-axial mass, often demonstrating a “CSF cleft sign” and adjacent reactive hyperostosis. The typical MRI signal intensity characteristics include an iso- to slightly hypointense signal compared to the cortex on T1-weighted imaging and an iso- to moderately hyperintense signal relative to the cortex on T2-weighted imaging. This is often accompanied by an area of central hypointensity (likely representing calcification) and surrounding vasogenic edema [1,18].

Fluid-attenuated inversion recovery sequences are particularly valuable for depicting peritumoral edema, with meningiomas typically displaying signal intensities ranging from isointense to hyperintense relative to the brain on fluid-attenuated inversion recovery imaging. T2* sequences (such as gradient echo and SWI) help illustrate intratumoral calcification. The phenomenon of “blooming,” secondary to intratumoral hemorrhage, is infrequent. Most meningiomas do not show restricted diffusion on diffusion-weighted imaging. T1-weighted postcontrast imaging reveals heterogeneous enhancement with a “dural tail sign” and extension into adjacent hyperostosis. Contrast administration is particularly beneficial in delineating “en plaque” meningiomas, which typically manifest as asymmetrically thickened sheets of enhancing duramater [1,18].

Magnetic Resonance Spectroscopy is a gold standard for advanced imaging diagnosing meningiomas. The characteristic features of these tumors include an increased choline peak (3.2 ppm), decreased creatine (3.0 ppm), and decreased N-acetyl aspartate. In some studies, lactate has been found more frequently in nonbenign meningiomas—those of WHO Grades II and III—but it is not a consistent marker of aggressive meningiomas. Lipids (Lip, 0.9/1.3 ppm) are typically regarded as markers of aggressive meningiomas [18,19]. Alanine (Ala, peak at 1.48 ppm) is often elevated. However, glutamate-glutamine (Glx, peak at 2.1-2.6 ppm) and glutathione (GSH, peak at 2.95 ppm) may serve as more specific potential markers [1,19].

The recent development of magnetic resonance elastography (MRE) provides an alternative to traditional MRI. MRE is an MRI-based technique that quantifies intratumoral stiffness by measuring the propagation of vibration-induced displacement and viscoelastic mechanical properties in targeted tissues. However, while MRE represents a promising development, it cannot be considered completely noninvasive due to the necessity of delivering shear force to the patient's brain [20,21].

The therapeutic approach for treating meningiomatosis encompasses surgery, radiation therapy, and stereotactic radiosurgery. Surgery is the preferred treatment for meningiomatosis. Chemotherapy is a treatment option for patients with inoperable or higher-grade tumors, particularly when disease recurrence is a concern postsurgery and radiation therapy. To date, chemotherapy has played only a minimal role in meningioma treatment [7,^11^,22]. The prognosis for patients with meningiomatosis is generally good, with morbidity and mortality rates. It does not differ significantly from the prognosis associated with solitary meningiomas [4,7].

In this case report, a 53-year-old female patient presented with complaints of a headache. CT and MRI examinations revealed multiple brain lesions due to meningiomatosis. Subsequent tissue biopsy identified these as meningothelial meningiomas, which is considered a rare occurrence in line with existing literature and our current understanding. This case displayed the possibility of meningiomatosis presenting as multiple brain lesions, particularly in elderly patients.

## Conclusion

Meningiomatosis represents a rare manifestation of meningiomas, with a higher incidence in women. Imaging is critical in confirming the diagnosis and describing the extent and mass effect on crucial cranial structures, which is vital for determining the appropriate therapies.

## Patient consent

A written consent was obtained from the patient for publication of this case and any accompanying images.
